# Are disease-related symptoms important to predicting developing diabetes from prediabetes?

**DOI:** 10.55730/1300-0144.5412

**Published:** 2022-04-10

**Authors:** Sibel ENGİN, Tolga AKKAN, Murat DAĞDEVİREN, Tijen ŞENGEZER, Mustafa ALTAY

**Affiliations:** 1 Department of Family Medicine, Keçiören Health Administration and Research Center, University of Health Sciences, Ankara, Turkey; 2 Department of Endocrinology and Metabolism, Keçiören Health Administration and Research Center, University of Health Sciences, Ankara, Turkey

**Keywords:** Diabetes symptoms checklist scale, prediabetes, symptom, type 2 diabetes mellitus

## Abstract

**Background/aim:**

There are not many studies conducted to detect and recognize the symptoms during the prediabetes period. In our study, we aimed to determine the symptoms that can be seen in prediabetes and diabetes and their prevalence and to determine the similarities and differences between the two groups.

**Materials and methods:**

Individuals who were diagnosed with prediabetes or diabetes, over the age of 18, literate, and accepted to collaborate were included in our study. The “Diabetes Symptoms Checklist Scale” was used by interviewing 321 participants, 161 prediabetic and 160 diabetic, face-to-face.

**Results:**

It has been found that the most common symptom in both the prediabetes and the diabetes group is “fatigue” (88.2% prediabetes, 89.4% diabetes). The symptoms seen in the dimensions of neurology and hyperglycemia are more common in individuals with diabetes than in individuals with prediabetes [neurology score: 1.85 ± 0.84 vs. 1.66 ± 0.64 (p = 0.02), respectively; hyperglycemia score: 2.39 ± 0.94 vs. 2.08 ± 0.83 (p = 0.002), respectively]. It was observed that the symptom burden increased in all subdimensions with the long duration of illness, being a female, not working, having a family history, and not doing exercise, and high fasting blood glucose and high HbA1c values. The level of education, family history, accompanying hyperlipidemia, neurology, and hyperglycemia symptoms are associated with diabetes; and it has been determined that cardiology symptoms are associated with prediabetes.

**Conclusion:**

Especially; during the follow-up of patients with prediabetes who have a low education level and diabetic family history and concomitant hyperlipidemia, there may be an increase in neurological and hyperglycemic symptoms at the point of development of type 2 diabetes. In this respect, we recommend that these factors, which we found to be predictive of diabetes compared to prediabetes, should be questioned more carefully during patient visits.

## 1. Introduction

The concept of prediabetes includes individuals who do not meet the sufficient criteria for a diagnosis of diabetes, but whose blood sugar is above normal limits. Prediabetes is an important risk factor for the development of type 2 diabetes mellitus (T2DM). In addition, the complication rate of 10%–40% in newly diagnosed T2DM patients at the time of diagnosis and the fact that macrovascular and microvascular complications associated with T2DM can start to develop from the prediabetes period indicate that this prediabetic period is not a quiet and innocent period [1]. Therefore, the ability to prevent or delay the development of T2DM and diabetes-related complications with early diagnosis increases the clinical importance of prediabetes diagnosis.

There are many symptoms seen during the natural course of prediabetes and T2DM disease and as a result of the complications they cause. Symptoms vary greatly from person to person and are influenced by many factors. While some of these symptoms are more specific to the disease, some symptoms may be nonspecific. Especially in the early period, more nonspecific and subtle symptoms can be encountered. In addition, there is not enough research and clear data on which diabetes symptoms are seen and how often they are encountered during the prediabetes period. All of these can cause delays in the diagnosis of diabetes.

In our study, we aimed to identify common symptoms in prediabetes and T2DM patients, compare these two periods in terms of symptom characteristics, and thus obtain new data that can guide the diagnosis of prediabetes and diabetes in an earlier period in clinical practice.

## 2. Materials and methods

### 2.1. Study protocol and sampling

The study is an analytical, cross-sectional study conducted between June 25, 2020–July 31, 2020. According to the power analysis, a minimum of 150 patients with prediabetes diagnoses and 150 patients with T2DM diagnoses were planned to be included in the study. Our study was conducted with the approval of the local ethics committee (2012-KAEK-15/2124). The study was carried out in accordance with the Helsinki Declaration and verbal and written consent was obtained from all participants.

T2DM and prediabetes patients diagnosed according to the American Diabetes Association (ADA) criteria and under follow-up were included in the study [2]. T2DM diagnosis; HbA1c ≥ 6.5% or fasting plasma glucose ≥126 mg/dL or 2nd-h glucose value ≥200 mg/dL in the oral glucose tolerance test or random plasma glucose ≥200 mg/dL with classical hyperglycemia symptoms was determined by the presence of at least one of the criteria. Prediabetes diagnosis; HbA1c 5.7%–6.4% or fasting plasma glucose 100–125 mg/dL or 2nd-h glucose value 140–199 mg/dL in the oral glucose tolerance test by the presence of at least one of the criteria [2]. During the study period, a total of 422 T2DM and prediabetes diagnosed individuals who applied to the clinics where the study was conducted were evaluated, and 321 adult participants, who were literate, had no psychiatric disability, and had accessible laboratory data, were included in the study. Patients with acute illness and Covid 19 infection in the last 2 weeks were not included in the study (Figure).

After obtaining verbal and written informed consent from the patients who accepted to participate in the study and met the current criteria, the following questionnaire forms were applied in an appropriate polyclinic room by face-to-face interview.

Participants’ age, gender, marital status, education level, occupation, monthly income level, employment status, how long they have been diagnosed with diabetes or prediabetes, family history, additional diseases, medications used for treatment, compliance with treatment, smoking and alcohol use, exercise and diet status, regular medical control status, current laboratory values and the presence of a chronic complication were evaluated with a case report form consisting of 19 items. Fasting blood glucose and HbA1c values of the patients were recorded from the hospital automation system and patient files.

The symptoms of the participants were evaluated with the Diabetes Symptoms Checklist Scale, which was developed by Grootenhuis et al. in 1994 and the validity and reliability study in Turkish was conducted by Terkeş and Bektaş in 2012 [3].

Diabetes Symptoms Checklist Scale is a Likert-type scale consisting of 33 questions with answer options ranging from 1 to 5 such as “none”, “a little”, “moderate”, “very”, “excessive”. It includes six subdimensions: neurology, psychology/fatigue, cardiology, ophthalmology, psychology/cognitive, and hyperglycemia.

### 2.2. Statistical analysis

The data obtained were analyzed with IBM SPSS 22.0 (IBM Corp. Released 2013. IBM SPSS Statistics for Windows, Version 22.0. Armonk, NY: IBM Corp.) program. Categorical data were expressed as numbers and percentages. The normal distribution of the data was examined with the Kolmogorov-Smirnov test and histogram, and since the data were normally distributed, they were expressed as mean and standard deviation. The student t-test was used to compare the normal distributed continuous data between two groups. Cronbach Alpha test was used to test the reliability of the applied scale data. Pearson’s correlation test was used for correlation analysis. Logistic regression analysis modeling was performed to predict diabetes risk according to prediabetes. Hosmer & Lemeshow test was performed in terms of model fit. A value of p < 0.05 was accepted for statistical significance.

## 3. Results

Two hundred and fifty-eight (80.35%) of the participants in the study are women; 63 of them (19.65%) were male. The average age was 56.1 (min: 29, max: 90). Age, gender, marital status, education level, employment status, occupation, monthly income, smoking and alcohol use, exercise, and diet status were similar between the two groups. When both groups were examined in terms of chronic diseases, it was found that hypertension, heart disease, and hyperlipidemia were significantly higher in the diabetes group compared to the prediabetes group (Table 1).

While 101 patients (62.7%) in the prediabetic group had a family history, 118 individuals (73.8%) in the diabetes patient group had a family history (p = 0.03). When the two groups were compared in terms of disease duration; the mean duration of illness was significantly higher in the diabetes group compared to the prediabetes group (113.57 months, 31.34 months, respectively; p < 0.001), while the patients with poor compliance with treatment were in the majority (48.7%) in the prediabetes group, those in the diabetes group were in the majority (42.5%) (Table 2).

When two groups are examined in terms of complications; while 1 patient in the prediabetes group had retinopathy (0.6%), 19 patients (11.9%) in the diabetes group had retinopathy (p < 0.001). Other complications were only present in the diabetes group (Table 2).

Considering the laboratory values; fasting blood glucose and HbA1c values were statistically significantly higher in the diabetes group (p < 0.001) (Table 2).

Considering the application results of the Diabetes Symptoms Checklist Scale; it was observed that the symptom that the patients experienced in both groups at the highest rate (88.2% of those with prediabetes, 89.4% of those with diabetes) was “fatigue” at any level (a little, moderate, very, excessive).

When the two groups were compared in terms of symptoms; it was found that the symptom of “difficulty in concentrating on a subject” was observed at a higher rate in individuals with prediabetes than in individuals with diabetes (54% and 42.5%) (p = 0.04). The diabetes group was found to be more symptomatic than the prediabetes group in terms of “the need to urinate frequently” and “burning pain in the legs during the day” symptoms (p < 0.001 and p = 0.03, respectively). The rates of other symptoms were similar between the two groups (p > 0.05) (Table 3).

When the data are analyzed at the level of the subdimensions of the scale; it was found that there was a statistically significant difference only in the scores obtained from the neurology and hyperglycemia subdimensions between the two groups. Individuals with prediabetes had a score of 1.66 ± 0.64 in the neurology subdimension, while the score of individuals with diabetes was 1.85 ± 0.84 (p = 0.02). In the hyperglycemia subdimension, the average score of individuals with prediabetes was 2.08 ± 0.83, while the average score of individuals with diabetes was 2.39 ± 0.94 (p = 0.002) (Table 4).

As the duration of illness, fasting blood glucose and HbA1c values increase; it was observed that symptom scores increased in all subdimensions (Table 5).

Education level, presence of family history, accompanying hyperlipidemia, neurology, and symptoms of hyperglycemia were determined as associated factors for type 2 diabetes as compared to prediabetes; cardiology symptoms were found to be associated with prediabetes as compared to diabetes (Table 6).

## 4. Discussion

In our study, it has been found that the most common symptom in both the prediabetes group and the diabetes group is “fatigue”, and the symptoms seen in the dimensions of neurology and hyperglycemia are more common in individuals with diabetes than in individuals with prediabetes. It was observed that the symptom burden increased in all subdimensions with the long duration of illness, being a female, not working, having a family history, not doing exercise, and high fasting blood glucose and high HbA1c values. The level of education, family history, accompanying hyperlipidemia, neurology, and hyperglycemia symptoms are associated with diabetes; and it has been determined that cardiology symptoms are associated with prediabetes.

Prediabetes is an important risk factor for macrovascular complications, especially cardiovascular disease [4]. Prediabetes is also an important risk factor for microvascular complications, and diabetic retinopathy, nephropathy, and neuropathy have also been detected in individuals with prediabetes [5–7]. In addition, autonomic dysfunctions such as bradycardia and erectile dysfunction have been found to be associated with prediabetes [8].

In the light of this information available in the literature, it was a result that we expected to find similar symptom burdens in prediabetes and diabetes groups. In our study, only symptoms related to neurology and hyperglycemia subdimensions were more common in diabetic patients. In a study by Adriaanse et al; when individuals with normal glucose metabolism and diabetic individuals are compared, it has been reported that diabetic individuals have higher scores in terms of neuropathic pain, emotional symptoms, and total symptom burden [9].

In our study, the highest scores in both groups were obtained from the psychology (fatigue) and hyperglycemia dimensions. In the study of Terkeş and Bektaş, it was found that individuals with diabetes experienced the most neurological and psychological (cognitive) symptoms [10]. In another study, Kumsar et al. found that individuals with type 2 diabetes experienced more hyperglycemia and psychological (fatigue) symptoms, similar to our study [11]. When the symptoms are examined separately in our study; it was determined that the most common symptom experienced by both groups was “fatigue”. In the study conducted by Kumsar et al. using the same scale, it was found that diabetic individuals experienced the symptoms of “get angry quickly”, “need to urinate frequently” and “fatigue” to an “excessive” degree [11]. In another study by Adriaanse et al.; “fatigue”, “dry mouth” and “drowsiness or dizziness” were found to be the most common symptoms experienced by type 2 diabetes patients [12]. These results can be explained by the fact that psychology (fatigue) symptoms are not only seen in glucose metabolism disorders and can be seen at high levels in the general population.

In addition, in our study, we found that the increase in disease duration was associated with increased symptom burden in all subdimensions. Again, increased HbA1c and fasting blood glucose values cause an increase in symptoms in all dimensions. This finding is a finding supported by many studies including Terkeş’s study and the studies of Kumsar et al. [3,9,11,13–16].

The positive family history of a patient who is under follow-up and known to be prediabetic is a risk factor for the development of type 2 diabetes. This is not a surprising result because diabetes is a disease with a strong genetic basis [17]. The fact that a low education level is associated with an increased risk of type 2 diabetes may be due to the fact that educated individuals are more conscious, more knowledgeable about their diseases and their treatments, and more inclined to read and research. Concomitant hyperlipidemia is one of the predictors of type 2 diabetes development because diabetes refers not only to the defect in carbohydrate metabolism but also to the defect in fat and protein metabolism [18]. Increased symptoms in neurology and hyperglycemia subdimensions were also found to be factors indicating the progression from prediabetes to type 2 diabetes. One of the most common microvascular complications of diabetes is neuropathy [19]. Along with the symptoms caused by neuropathy, increased hyperglycemic symptoms, which are a result of impaired glucose metabolism during the transition from prediabetes to diabetes, may also alert the physician to the development of diabetes. In this context, detailed questioning of the neurological and hyperglycemic symptoms of the patients by the physician may be a guide in preventing the development of diabetes.

Symptoms in the cardiology subdimension were associated with prediabetes. This may be due to the fact that patients with type 2 diabetes are under stricter cardiological follow-up and are using treatments that suppress symptoms of the cardiovascular system such as beta-blockers. In addition, reactive hypoglycemia symptoms, which can be seen more frequently in the prediabetes patients, may be confusing with symptoms in the cardiology subdimension.

Our study is the first study comparing symptoms of prediabetes and type 2 DM in a wide sociodemographic data scale in Turkey. But the fact that the study was conducted during the Covid-19 pandemic process may have led to a bias in terms of patient application and symptom evaluation that we could not intervene. In addition, the inclusion of menopausal women may have confused menopausal symptoms with other symptoms. The limitations of our study are that some tests (such as vitamin D level, and vitamin B12 level) were not performed in the evaluation of some nonspecific symptoms, only the patient’s statement was taken into account, and body mass index was not evaluated.

In the light of all this information, during the follow-up of patients diagnosed with prediabetes, low education level, diabetic family history, and concomitant hyperlipidemia, there may be an increase in neurological and hyperglycemic symptoms at the point of development of type 2 diabetes. In this respect, it may be a warning for the physician to carefully question these factors, which we found to be predictive of diabetes compared to prediabetes, during patient visits. However, further studies are needed to obtain clearer results.

## Figures and Tables

**Figure:**
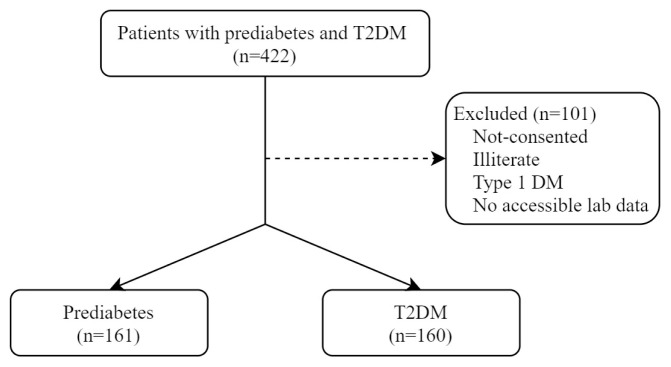
Determination of patient groups.

**Table 1:** General demographic characteristics of the study group.

	Prediabetes (n = 161)	Type 2 DM (n = 160)	p
Age – year	55.2 ± 11.71	57.06 ± 9.81	>0.05*
Gender – n (%)			>0.05‡
Female	134 (83.2)	124 (77.5)	
Male	27 (16.8)	36 (22.5)	
Marital status – n (%)			>0.05‡
Nonmarried	36 (22.4)	35 (21.9)	
Married	125 (77.6)	125 (78.1)	
Level of education– n (%)			>0.05‡
Mid-school and lower	130 (80.7)	126 (78.8)	
High School	19 (11.8)	29 (18.1)	
University and higher	12 (7.5)	5 (3.1)	
Employment Status – n (%)			>0.05‡
Unemployed	143 (88.8)	139 (86.9)	
Full-time employed	18 (11.2)	21 (13.1)	
Occupancy – n (%)			>0.05‡
Officer	5 (3.1)	7 (4.4)	
Worker	5 (3.1)	5 (3.1)	
Self-employed	5 (3.1)	11 (6.9)	
House wife	121 (75.2)	112 (70)	
Retired	20 (12.4)	23 (14.4)	
Other	5 (3.1)	2 (1.3)	
Monthly income – n (%)			>0.05‡
Below minimum wage	35 (21.7)	45 (28.2)	
Minimum wage	43 (26.7)	46 (28.7)	
2500–5000 TL	70 (43.5)	53 (33.1)	
5000 TL and higher	13 (8.1)	16 (10)	
Smoking – n (%)			>0.05‡
Nonsmoker	143 (88.8)	134 (83.8)	
Smoker	18 (11.2)	26 (16.2)	
Alcohol – n (%)			>0.05‡
Nondrinker	159 (98.8)	157 (98.1)	
Drinker	2 (1.2)	3 (1.9)	
Diet – n (%)			>0.05‡
Nondoing	127 (78.9)	123 (76.9)	
Doing	34 (21.1)	37 (23.1)	
Exercise– n (%)			>0.05‡
None	92 (57.1)	109 (68.1)	
Irregular	46 (28.6)	34 (21.3)	
Regular	23 (14.3)	17 (10.6)	
Chronic disease history – n (%)			
Hypertension	74 (46)	92 (57.5)	**0.04** ‡
Cardiovascular disease	17 (10.6)	31 (19.4)	**0.03** ‡
Obesity	24 (14.9)	37 (23.1)	>0.05‡
Kidney disease	4 (2.5)	5 (3.1)	>0.05‡
Thyroid disease	34 (21.1)	30 (18.8)	>0.05‡
Hyperlipidemia	30 (18.6)	64 (40)	**<0.001** ‡
Neurological disease	7 (4.3)	1 (0.6)	
Psychiatric disease	4 (2.5)	6 (3.8)	>0.05‡
Ophthalmological disease	35 (21.7)	44 (27.5)	>0.05‡
Chest disease	23 (14.3)	31 (19.4)	>0.05‡
Other chronic diseases	20 (12.4)	9 (5.6)	**0.03** ‡

Abbreviations; TL: Turkish lira, DM: Diabetes Mellitus

* Student’s t-test was used.

‡ Pearson’s chi-square/Fisher’s exact test was used.

**Table 2:** Comparison of study groups’ data on primary diseases.

	Prediabetes (n = 161)	Type 2 DM (n=160)	p
Type 2 DM family story – n (%)	101 (62.7)	118 (73.8)	**0.03** ‡
Duration of illness – month	31.34 ± 40.82	113.57 ± 87.25	**<0.001** *
Periodic medical check – n (%)			>0.05‡
No	70 (43.5)	84 (52.5)	
Yes	91 (56.5)	76 (47.5)	
Drug usage – n (%)			
Noninsulin Antidiabetic agent	44 (27.3)	142 (88.8)	**<0.001** ‡
Insulin	0	30 (18.8)	
Treatment compliance– n (%)			**<0.001** ‡
Poor	78 (48.4)	41 (25.6)	
Medium	44 (27.3)	51 (31.9)	
Good	39 (24.2)	68 (42.5)	
Complications of diabetes– n (%)			
Cardiovascular diseases	0	4 (2.5)	
Diabetic foot	0	3 (1.9)	
Retinopathy	1 (0.6)	19 (11.9)	**<0.001** ‡
Neuropathy	0	9 (5.6)	
Nephropathy	0	7 (4.4)	
Fasting blood glucose– mg/dL	101.34 ± 9.72	157.73 ± 53.99	**<0.001** *
HbA1c – %	5.81 ± 0.40	7.92 ± 1.73	**<0.001** *

Abbreviations; DM: Diabetes Mellitus, HbA1c: Hemoglobin A1c

‡ Pearson’s chi-square/Fisher’s exact test was used.

* Student’s t-test was used.

**Table 3:** Comparison of the groups in terms of the frequency of symptoms*.

	Prediabetes (n = 161)	Type 2 DM (n = 160)	p‡
1. Fatigue	142 (88.2)	143 (89.4)	>0.05
2. Pain in the calves when walking	94 (58.4)	100 (62.5)	>0.05
3. Numbness (loss of sensation) in the feet	58 (36)	67 (41.9)	>0.05
4. A general feeling of exhaustion/exhaustion	124 (77)	121 (75.6)	>0.05
5. Shortness of breath at night	33 (20.5)	35 (21.9)	>0.05
6. Drowsiness or dizziness	96 (59.6)	103 (64.4)	>0.05
7. Difficulty concentrating on a subject	87 (54)	68 (42.5)	**0.04**
8. Emotional changes	127 (78.9)	123 (76.9)	>0.05
9. Numbness in the hands (loss of sensation)	96 (59.6)	80 (50)	>0.05
10. Blurred vision that does not go away even when wearing glasses	54 (33.5)	57 (35.6)	>0.05
11. Tingling in arms and legs at night	63 (39.1)	74 (46.3)	>0.05
12. Excessive thirst	72 (44.7)	87 (54.4)	>0.05
13. Palpitations	64 (39.8)	64 (40)	>0.05
14. Impaired vision	76 (47.2)	70 (43.8)	>0.05
15. Burning pain in the calves at night	89 (55.3)	93 (58.1)	>0.05
16. Dry mouth	111 (68.9)	124 (77.5)	>0.05
17. Increased fatigue during the day	104 (64.6)	104 (65)	>0.05
18. Lightning flashes or black spots in the field of vision	93 (57.8)	97 (60.6)	>0.05
19. Anger before eating	55 (34.2)	63 (39.4)	>0.05
20. Feeling exhausted when woke up in the morning	109 (67.7)	111 (69.4)	>0.05
21. Sudden stinging pain in the legs under the knee and feet	54 (33.5)	59 (36.9)	>0.05
22. Sometimes clear, sometimes blurred vision	90 (55.9)	86 (53.8)	>0.05
23. The need to urinate frequently	93 (57.8)	121 (75.6)	**0.001**
24. Pain in the chest or heart area	58 (36)	67 (41.9)	>0.05
25. Burning pain in the legs during the day	44 (27.3)	62 (38.8)	**0.03**
26. Tingling sensation and numbness in hands or fingers	92 (57.1)	85 (53.1)	>0.05
27. Get angry quickly	114 (70.8)	110 (68.8)	>0.05
28. Sudden deterioration in vision	33 (20.5)	30 (18.8)	>0.05
29. A different feeling in the feet and legs below the knee when touched	50 (31.1)	51 (31.9)	>0.05
30. Difficulty breathing during physical activity	90 (55.9)	85 (53.1)	>0.05
31. Feeling dizzy in the head (difficulty in thinking clearly)	84 (52.2)	78 (48.8)	>0.05
32. Drinking too much liquid (all kinds of drinks)	74 (46)	91 (56.9)	>0.05
33. Difficulty in concentrating	79 (49.1)	66 (41.3)	>0.05
34. Tingling sensation and numbness in the area of the legs below the knee and in the feet	64 (39.8)	60 (37.5)	>0.05

* The rates of prediabetes and diabetes group responding to the scale questions as “a little”, “moderate”, “very”, or “excessive” are shown.

Abbreviations; DM: Diabetes Mellitus

‡ Pearson’s chi-square/Fisher’s exact test was used.

**Table 4:** Comparison of subdimensions with which questions are grouped. *

	Prediabetes (n = 161)	Type 2 DM (n = 160)	Cronbach’s Alpha	p‡
Psychology (exhaustion)	2.29 ± 0.79	2.33 ± 0.84	0.81	>0.05
Psychology (cognitive)	1.86 ± 0.68	1.86 ± 0.67	0.68	>0.05
Neurology	1.66 ± 0.64	1.85 ± 0.84	0.79	**0.02**
Cardiology	1.75 ± 0.55	1.73 ± 0.57	0.77	>0.05
Ophthalmology	1.49 ± 0.53	1.54 ± 0.66	0.81	>0.05
Hyperglycemia	2.08 ± 0.83	2.39 ± 0.94	0.71	**0.002**

Abbreviations; DM: Diabetes Mellitus

* Points; it was calculated by dividing the total scores of the answers given to the questions in the relevant subdimension by the number of questions in the subdimension.

‡ Student’s t-test was used.

**Table 5:** Correlation analysis between demographic data and subdimension symptoms in the whole study group.*

	Neurology		Psychology (exhaustion)		Psychology (cognitive)		Cardiology		Ophthalmology		Hyperglycemia	
	*r*	*p*	*r*	*p*	*r*	*p*	*r*	*p*	*r*	*p*	*r*	*p*
**FBG**	**0.21**	**<0.01**	0.06	0.26	0.01	0.90	0.06	0.29	**0.17**	**<0.01**	**0.24**	**<0.01**
**HbA1c**	**0.24**	**<0.01**	0.09	0.09	0.07	0.24	0.08	0.14	**0.20**	**<0.01**	**0.22**	**<0.01**
**Disease period**	**0.34**	**<0.01**	0.19	<0.01	0.12	0.04	0.19	<0.01	**0.25**	**<0.01**	**0.30**	**<0.01**

Abbreviations; FBG: Fasting blood glucose, HbA1c: Hemoglobin A1c

* Pearson’s correlation test was used.

**Table 6:** Logistic regression analysis to investigate independent risk factors predicting diabetes according to prediabetes.

Risk factor	B (SE)	OR (%95 CI)	p
Age	−0.002 (0.014)	0.99 (0.97–1.03)	>0.05
Gender			
Female (RC)		1	
Male	0.530 (0.383)	1.7 (0.80–3.60)	>0.05
Level of education			
Mid-school and lower (RC)		1	
High School	**0.885 (0.408)**	**2.42 (1.09**–**5.39)**	**0.03**
University and higher	−0.907 (0.701)	0.4 (0.10–1.60)	>0.05
Monthly income			
Below minimum wage (RC)		1	
Minimum wage	0.114 (0.354)	1.12 (0.56–2.24)	>0.05
2500–5000 TL	−0.442 (0.354)	0.64 (0.32–1.29)	>0.05
5000 TL and higher	0.342 (0.571)	1.41 (0.46–4.31)	>0.05
Family history of type 2 DM			
No (RC)		1	
Yes	**0.640 (0.275)**	**1.90 (1.11**–**3.25)**	**0.02**
Exercise			
None (RC)		1	
Irregular	−0.308 (0.307)	0.74 (0.40–1.34)	>0.05
Regular	−0.414 (0.412)	0.66 (0.30–1.49)	>0.05
Diet			
Not doing (RC)		1	
Doing	0.085 (0.321)	1.09 (0.58–2.04)	>0.05
Hypertension			
No (RC)		1	
Yes	0.251 (0.297)	1.29 (0.72–2.30)	>0.05
Obesity			
No (RC)		1	
Yes	0.190 (0.336)	1.21 (0.63–2.34)	>0.05
Hyperlipidemia			
No (RC)		1	
Yes	**1.131 (0.304)**	**3.10 (1.71**–**5.62)**	**<0.001**
Psychology (exhaustion)	−0.075 (0.222)	0.93 (0.60–1.43)	>0.05
Psychology (cognitive)	−0.172 (0.230)	0.84 (0.54–1.32)	>0.05
Neurology	**0.670 (0.256)**	**1.95 (1.18**–**3.23)**	**0.01**
Cardiology	**−** **0.848 (0.381)**	**0.43 (0.20**–**0.90)**	**0.03**
Ophthalmology	−0.094 (0.261)	0.91 (0.55–1.52)	>0.05
Hyperglycemia	**0.423 (0.164)**	**1.53 (1.11**–**2.10)**	**0.01**

* R^2^ = 0.23

** Hosmer & Lemeshow test p = 0.42

Abbreviations; SE: Standard Error, OR: Odds Ratio, CI: Confidence Interval, RC: Reference category.
